# Effectiveness of government policies in response to the first COVID-19 outbreak

**DOI:** 10.1371/journal.pgph.0000242

**Published:** 2022-04-13

**Authors:** Theologos Dergiades, Costas Milas, Elias Mossialos, Theodore Panagiotidis

**Affiliations:** 1 Department of International & European Studies, University of Macedonia, Thessaloniki, Greece; 2 Management School, University of Liverpool, Liverpool, United Kingdom; 3 Department of Health Policy, London School of Economics and Political Science, London, United Kingdom; 4 Department of Economics, University of Macedonia, Thessaloniki, Greece; Public Health Foundation of India, INDIA

## Abstract

This paper assesses the quantitative impact of government interventions on deaths related to the first COVID-19 outbreak. Using daily data for 32 countries and relying on the stringency of the conducted policies, we find that the greater the *strength* of government interventions at an *early stage*, the more effective these are in slowing down or reversing the growth rate of deaths. School closures have a significant impact on reducing the growth rate of deaths, which is less powerful compared to the case where a number of policy interventions are combined together. These results can be informative for governments in responding to future pandemics.

## 1. Introduction

As evident by the tremendous media attention, the COVID-19 pandemic has triggered severe social and economic costs. The Financial Times, for instance, has a dedicated website which provides free access to its analysis (https://www.ft.com/coronavirusfree). At the time of writing, there were around 353 million confirmed cases of COVID-19 infections and 5.6 million deaths (https://covid19.who.int/). Lessons learned from previous pandemics imply a huge impact on economic activity. Barro *et al*. [1] use data for 43 countries to find that the ‘Spanish flu’ of the 1918–1920 period generated real per capita GDP declines of 6% for countries on average. Using data stretching back to the 14th century for France, Germany, Italy, the Netherlands, Spain, and the UK, Jordà *et al*. [2] show that pandemics depress the real rate of interest for years after a pandemic, perhaps as many as 40 years (wars do not have such effects).

To bring down COVID-19-related infections and deaths in the pandemic thus far, governments have responded with a number of interventions. Among others, Cowling *et al*. [3] show that non-pharmaceutical interventions (including border restrictions, quarantine and isolation, distancing, and changes in population behavior) were associated with reduced transmission of COVID-19 in Hong Kong. Using data for Germany, Hartl *et al*. [4] find a reduction in the growth rate of COVID-19, seven days after the implementation of containment policies on 13 March 2020 and again eight days after the implementation of further measures on 22 March 2020. Chen and Qiu [5] rely on a dynamic panel epidemiological model of nine countries to show that interventions like mask wearing and centralized quarantine can replace the costly, in economic terms, national lockdown without significantly heightening the epidemic peak. Hsiang *et al*. [6] use panel regression analysis to find that interventions prevented or delayed around 530 million COVID-19 infections across six countries (China, South Korea, Iran, Italy, France, and the United States). Chudik *et al*. [7] rely on an epidemiological model for a number of Chinese provinces and ten countries to find that it takes about 21 days from infection to recovery (or death) rather than the 14 days typically assumed in designing quarantine policies.

As the first wave of the COVID-19 outbreak appears to be easing in many countries around the globe, it is extremely crucial to provide an initial quantitative assessment of the impact that ongoing government interventions have had on controlling the pandemic. As noted by a recent communication news article in Nature [8], “*working out the effectiveness of the measures implemented worldwide to limit the coronavirus’s spread is now one of scientists’ most pressing questions*”. The pressing issue of the efficacy of government interventions could be further addressed by the following questions: First, can a prompt and strict government response curb the mortality curve of the epidemic? Second, does the severity of interventions affect (and by how much) the subsequent evolution of the growth rate of deaths? Third, under uncontrolled pandemic conditions, can the shift to harsher measures lower (and by how much) the growth rate of deaths? Providing this information should be vital to governments as they attempt to design strategies to return to a ‘new normal’ and, at the same time, prevent further waves of the COVID-19 outbreak.

This paper attempts to address these critical questions in a quantitative manner. To do so, we first define two critical concepts: (i) the *strength* of the policies and (ii) the *early stage* implementation of these policies. In particular, we define the policy *strength* of any certain day as the average value of the Government Response Tracker index (developed by the University of Oxford) for the preceding 14 days (Lauer *et al*. [9] estimate that the incubation period of the virus is 14 days). *Early stage* refers to any day that precedes or is equal to the first observed day in which the number of confirmed deaths is at least five (both definitions are further discussed in section 5). Considering these concepts, we form three hypotheses of interest. “Speed is of the essence” is our first hypothesis. That is, if COVID-19 policy measures are effective, then the *strength* of these measures taken at an *early stage* should be related positively to the probability of attaining a statistically insignificant increasing trend in deaths attributable to COVID-19. Insignificant increasing trend implies that the trend may be: i) positive and insignificant, ii) negative and insignificant or iii) negative and significant. “Stringency matters” is our second hypothesis. Upon realization of a positive significant trend in deaths, and under the assumption that the COVID-19 related policy measures taken at an *early stage* have been effective, then the *strength* of these measures should still inversely affect the trend slope shaped by the COVID-19 deaths. “Speed of adjustment matters” is our third hypothesis. As countries that initially responded with “low *strength*” policy measures progressively increase the *strength* of their response, a significant reduction in the trend slope should take place after a certain breakpoint in time. Thus, for countries with a break in the trend slope, if COVID-19 policy measures are effective then the difference in the *strength* of the policy measures (between the break time and the *early stage*) should be related inversely to the difference of the slopes in the observed trends (after and before the break).

To examine these hypotheses, we use daily data on COVID-19 related deaths for 32 countries. We rely on the Perron and Yabu [10] statistical test to endogenously determine a break on the linear trend of the logarithm of deaths per country. We also estimate the slope of this trend based on the work of Perron and Yabu [11]. We then assess the impact of government interventions on the trend slope shaped by the daily number of deaths. Our results suggest that a unit increase in the *strength* of the overall policy measures (school closures) at an *early stage* raises the probability of attaining an insignificant increasing trend in deaths by 1.1% (0.7%). We also find that a unit increase in the overall policy measures (school closures) at an *early stage* lowers the growth rate of deaths by 0.2% (0.1%). Finally, governments that initially respond with “*low strength*” policy measures but progressively increase the *strength* of their response, succeed in slowing down or reversing the growth rate of deaths after a certain breakpoint in time. In particular, a unit increase in the adjustment of the overall policy (school closures) between the breakpoint in time and the early stage lowers the difference in the growth rate of deaths (observed after and before the breakpoint) by 0.3% (0.1%). Therefore, we find that the greater the *strength* of government interventions at an *early stage*, the more effective these are in slowing down or reversing the growth rate of deaths. Additionally, the evidence suggests that school closures alone, have a significant impact–albeit one which is less powerful in reducing the growth rate of deaths than that achieved by pooling together a number of government interventions. Overall, governments can use some of the results of this paper to respond to future pandemics as are likely to emerge.

The paper proceeds as follows: section 2 discusses the data; section 3 provides the theoretical motivation; section 4 describes the methodology; section 5 presents the empirical strategy and model estimates; section 6 presents robustness checks and, section 7 concludes.

## 2. Data

We focus on 32 countries that have been affected by the pandemic crisis and use daily data on the total number of confirmed deaths attributed to COVID-19. Our sample covers the January 1^st^, 2020 to April 30^th^, 2020 period. The accuracy of official confirmed cases of COVID-19-related infections is limited by how effectively a country is testing people to confirm cases and accurately reporting results. For example, Germany and South Korea have been much more aggressive in testing and confirming infections than other countries. Manski and Molinari [12] note the problem with missing data on confirmed cases of COVID-19 and rely on partial identification techniques to estimate infection rate bounds for two states in the United States (Illinois and New York) and Italy. With this in mind, we focus on deaths related to COVID-19. The raw data on the daily deaths come from the European Centre for Disease Prevention and Control (ECDC, https://cutt.ly/pyqHlRZ). The sample countries along with their ISO alpha-2 codes are: Argentina (AR), Austria (AT), Belgium (BE), Brazil (BR), Canada (CA), Chile (CL), China (CN), Denmark (DK), Egypt (EG), France(FR), Germany (DE), Greece (GR), Indonesia (ID), Iran (IR), Ireland (IE), Israel (IL), Italy (IT), Japan (JP), South Korea (KR), Malaysia (MY), the Netherlands (NL), Norway (NO), Panama (PA), Philippines (PH), Portugal (PT), Saudi Arabia (SA), Spain (ES), Sweden (SE), Switzerland (CH), Turkey (TR), United Kingdom (GB) and United States (US). For the case of France, due to the late and at the same time cumulative report of deaths from retirement homes and assisted living facilities we use the officially reported deaths in hospitals (https://bit.ly/32MTWFs). Similarly, for the case of China, we ignore the late and cumulative reported number of deaths that took place in late April.

**Fig 1:**
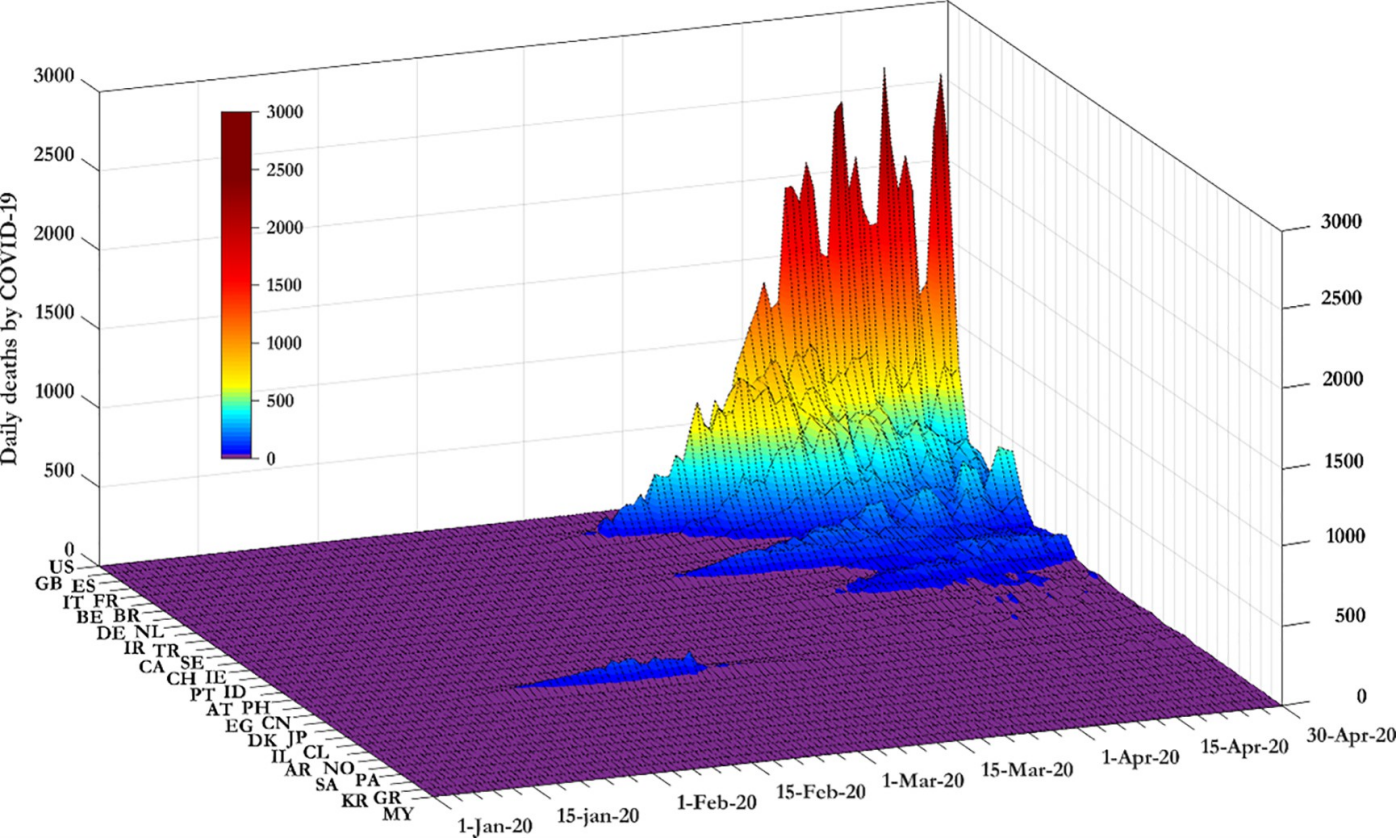
Confirmed deaths attributed to COVID-19 across countries.

Fig 1 reports the data in levels. We present that data in levels as Romano *et al*. [13] show that when COVID-19 related data are presented in a linear scale (compared to the log scale) eases the public to understand better the underlying information, improving this way the level of worry about the pandemic or even to affect public policy preferences. From Fig 1, daily deaths in China reached a peak in mid-February before dropping again (isolated blue surface in the purple area). Daily deaths in Italy reached a peak in late March 2020. Daily deaths in Spain, France and the UK reached a peak in early April 2020.

**Fig 2:**
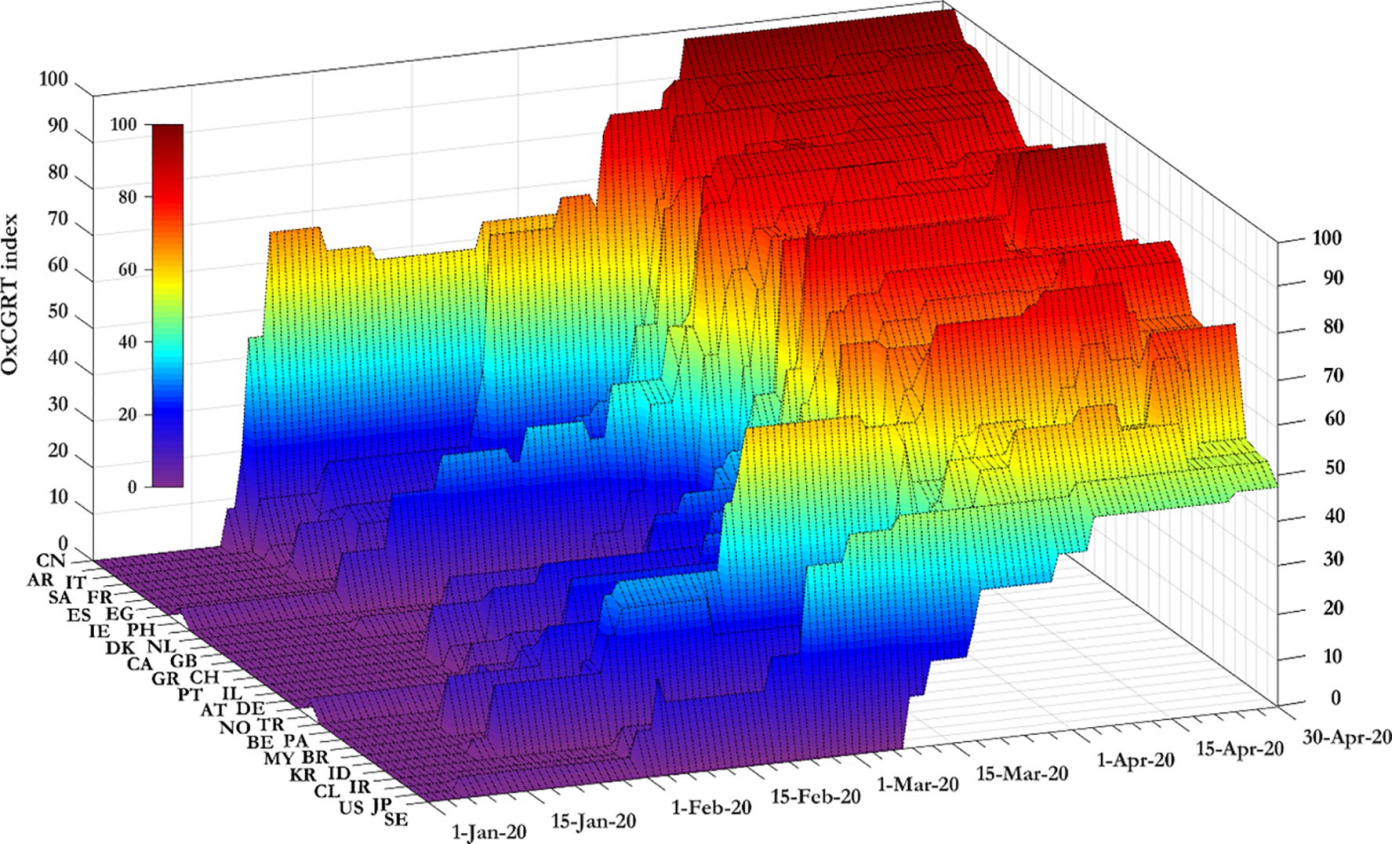
The OxCGRT index across countries.

Deaths due to COVID-19 forced governments to implement a range of policies to control the spread of the virus. The Blavatnik School of Government of the University of Oxford compiles the Oxford COVID-19 Government Response Tracker (OxCGRT) index [14], which quantifies the stringency of the conducted policies across countries. The value of the index on any given day in any given and country comes from the average of nine sub-indices (school closures, workplace closures, cancelation of public events, restrictions on gathering size, closure of public transport, stay at home requirements, restrictions on internal movement, restrictions on international travel, and public information campaign), each taking a value between 0 and 100 [14]. The raw data of the index for the sample countries (https://cutt.ly/mylfYBc) is shown in Fig 2.

From Fig 2, stringency measures in China increase steeply in late January 2020. Among other countries, stringency measures in Italy increase significantly in late February 2020. Then follow Spain and France, the UK and the US (notice that the left horizontal axes in Figs 1 and 2 report the sample countries in a different order. We do this to optimize the information content communicated by each Fig).

## 3. Theoretical motivation

The rationale for the structure of the estimated specifications in Section 5 can be deployed by linking the framework of a reduced-form econometric model, commonly implemented in economics to assess the impact of various conducted policies on target variables, and a typical epidemiological model. The use of reduced-form econometric specifications in epidemiology is not new. These specifications may prove quite informative when the purpose of the analysis is not to describe the mechanism of disease transmission, but rather to evaluate the impact of disease-related interventions on the number of cases or deaths [6]. The main advantage of reduced-form econometric modeling is that it permits sufficient inference on the total effectiveness of policy interventions without the need to provide input for essential epidemiological parameters that may be uncertain or even unknown.

Along the same lines, Hsiang *et al*. [6] rely on a typical Susceptible-Infected-Recovered (SIR) model to focus on the initial phase of the outbreak where the susceptible individuals in the limit approximate the population and the infections increase exponentially. Hsiang *et al*. [6] show that the initial system of equations is reduced to a simple first-order differential equation, which describes the change of infections in the beginning of the outbreak. The general solution for the number of infections at time t (I(t)) is:

$$I(t)=I(0)e^{gt},$$ (1)

where I(0) is the number of infections at the start of the process, e is the Naperian base and g is the growth rate of infections at each unit of time.

By calculating the natural logarithm for the ratio of the general solution over two sequential points in time (e.g. $t_{2}$ and $t_{1}$), it is easily shown that the growth rate of infections is equal to the difference of two principal parameters of the SIR model:

$$\log(I(t_{2})-\log(I(t_{1}))=g=\beta -\lambda ,$$ (2)

where β is the transmission rate of the disease and λ is the recovery rate. In the absence of vaccination and other government interventions (e.g. social distancing measures which affect β or health system investments that affect λ), the growth rate remains time-invariant. Under the realistic hypothesis that the very short-run investments on the health system do not alter λ, the growth rate of infections becomes time-dependent only when disease anti-contagion policies are implemented to the susceptible population. In other words, the growth rate of infections may be expressed as a function of the time-varying anti-contagion policies. Assuming a typical linear functional form, the growth rate of infections can be described by the following reduced form econometric specification:

$$\Delta \log(I(t))=\phi_{0}+\phi_{1}P(t)+u(t),$$ (3)

where $\phi_{0}$ is the growth rate of infections in the absence of social distancing policies, $\phi_{1}$ is the expected impact of these policies on the growth rate of infections, P(t) is a measure of stringency for the conducted social distancing policies at time t and u(t) is a normally distributed stochastic process with constant mean and constant variance.

Eq (3) describes the infection process for a disease and reveals that social distancing policies have a contemporaneous effect on the growth rate of the process. An emerging issue is whether the same process can be applied to model the growth rate of the resulting deaths. As there is a time distance between infection and death, the number of deaths at time t(D(t)) is proportional to the number of infections at an earlier time t−h. Hence, following Amaro *et al*. [15], the number of deaths at time t can be defined as:

D(t)=ξI(t−h), (4)

where ξ is the proportion of the infected individuals that finally die from the disease and h is the time horizon that separates infection from death. Given Eq (4), it can be shown that the growth rate of deaths at time t is equal to the growth of infections at time t−h:

Δlog(D(t))=Δlog(I(t−h)). (5)

In the presence of social distancing policies, Eqs (3) and (5) indicate that these may affect the growth rate of infections instantly (through the transmission parameter β), and impact the growth of deaths only after h time horizons. Thus, the reduced form econometric specification is:

$$\Delta \log(D(t))=\rho_{0}+\rho_{1}P(t-h)+z(t),$$ (6)

where $\rho_{0}$ is the growth rate of deaths in the absence of anti-contagion policies, $\rho_{1}$ is the average impact of the anti-contagion policies on the growth of deaths and z(t) is the error term. Eq (6) signals that the growth rate of deaths at time t depends on the anti-contagion policies implemented h time horizons in the past. As such, econometric specifications which ignore this structure can lead to misleading inferences.

## 4. Methodology

Epidemic curves consist of time-series observations showing the number of cases or deaths. The epidemiological stylized facts along with the respective theoretical underpinnings suggest that the level of an epidemic curve increases exponentially with time, while the log-level of the curve is approximately a linear function of time [16]. Hence, since our focus is on the log-level of deaths, the linear testing frameworks of Perron and Yabu [10, 11] come as a natural choice. In particular, Perron and Yabu [10] test for a break in the slope of a trend function of a series without prior information on whether the noise component is integrated of order one or a stationary process. For an autoregressive error component of order one (The extension for higher autoregressive structures is analytically discussed in [10]), we assume the following data-generated process for the variable $y_{t}$:

$$y_{t}=x_{t}^{\prime }w+u_{t},\;and\;u_{t}=au_{t-1}+e_{t},$$ (7)

where t=1,…,T, $e_{t}$ is an *i*.*i*.*d*. process with zero mean and variance $\sigma^{2}$, and **x**_*t*_ and **w** are (r×1) vectors containing the deterministic components of the series depending on the adopted specification and the unknown parameters, respectively. It is also assumed that $u_{0}$ is a finite scalar with a being in the range of (‐1,1], allowing this way the error component to be either stationary or integrated of order one. For our purpose, the specification of interest is model II (break in the trend) in Perron and Yabu [10]. Therefore, $x_{t}=(1,\;t,\;DT_{t}{)}^{\prime }$, t is a simple time trend, $DT_{t}=I(t>T_{1})(t‐T_{1})$ (where $T_{1}=[k_{1}T]$ is the break date at some $k_{1}\in (0,1)$ and I(.) is an inidcator function) and $w=(b_{0},\;b_{1},\;b_{2}{)}^{\prime }$. Hence, we focus our interest on testing the hypothesis **Rw** = g where **R** is a full rank matrix and **g** is a (q×1) vector of q restrictions; in this case, we focus on the significance of the $b_{2}$ coefficient.

When a=1, its approximation through OLS and the use of quasi-feasible generalized least squares (FGLS) to obtain estimates for **w** leads to a Wald statistic $W_{F}(k_{1})$ that no longer has a chi-square limit distribution. Hence, Perron and Yabu [10] propose a super-consistent estimate of a through the following truncation:

$${\hat{a}}_{MS}=\{\begin{array}{c} {\hat{a}}_{M}\;if\;|{\hat{a}}_{M}-1|>T^{-0.5}\\ 1\;if\;|{\hat{a}}_{M}-1|\leq T^{-0.5} \end{array}\},$$ (8)

where ${\hat{a}}_{M}=\hat{a}+C(\hat{\tau }){\hat{\sigma }}_{a}$ with $\hat{a}$ and ${\hat{\sigma }}_{a}$ the OLS estimate and the respective standard error and $C(\hat{\tau })$ an indicator function of the $\hat{\tau }$ ratio. In this case, ${\hat{a}}_{M}$ is the Roy and Fuller [17] coefficient correction for the biased estimate of $\hat{a}$ through OLS. For the case of a known break date, Perron and Yabu [10] show that the Wald statistic $W_{FMS}(k_{1})$ based on the FGLS with ${\hat{a}}_{MS}$ has a chi-square distribution irrespective of stationarity issues.

For the case of an unknown break, Perron and Yabu [10] propose the following $Exp‐W_{FMS}$ statistic estimated for all permissible break dates:

$$Exp‐W_{FMS}=\log[T^{-1}\sum_{K}\exp(0.5*W_{FMS}({k^{\prime }}_{1}))],$$ (9)

where $K=(k_{1}^{\prime };\varepsilon \leq k_{1}^{\prime }\leq 1‐\varepsilon )$ for ε>0 with $k_{1}^{\prime }$ being the break fraction in the sample and ε a trimming parameter. The problem with the limit distribution of $Exp‐W_{FMS}$ statistic is that it differs for the cases of an integrated of order one or a stationary process. Consequently, Perron and Yabu [10] identify the limit distributions and provide asymptotic critical values for different levels of the trimming parameter ε. Although the limit distributions are different for the two cases above, the critical values for the respective quantiles are quite similar. Hence, Perron and Yabu [10] propose to use the larger critical value.

Once the break in trend is identified, Perron and Yabu [11] propose an approach to estimate and test the significance of the linear trend in a series $y_{t}$. The approach is again robust to the presence of an integrated or stationary error component. In particular, assuming that in Eq (7) the **x**_*t*_ vector reduces to $x_{t}=(1,\;t{)}^{\prime }$ (in which case **w** is a 2×1 vector), the recommended procedure by Perron and Yabu [11] is as follows: (i) obtain the OLS residuals ${\hat{u}}_{t}$, (ii) find the Weighted Symmetric Least-Squares estimate ${\hat{a}}_{W}$ for the autoregressive parameter a as described in Roy and Fuller [17], (iii) find the value of ${\hat{a}}_{M}$ by ${\hat{a}}_{M}={\hat{a}}_{W}+C({\hat{\tau }}_{W}){\hat{\sigma }}_{a}$ (set $\tau_{pct}$ to -1.96; see [11]), (iv) calculate the trancated estimate ${\hat{a}}_{MS}$ based on Eq (8) and finally, (v) apply, using ${\hat{a}}_{MS}$, the GLS approach to get the estimate for the slope coefficient ${\hat{b}}_{1}$. The significance of ${\hat{b}}_{1}$ is assessed by the resulting standard t-statisitc. Overall, both approaches of Perron and Yabu [10, 11] are robust to heteroskedastic errors. Thus, their application, may assist in reducing the effects of error in the measurement of data.

## 5. Empirical strategy and findings

**Fig 3:**
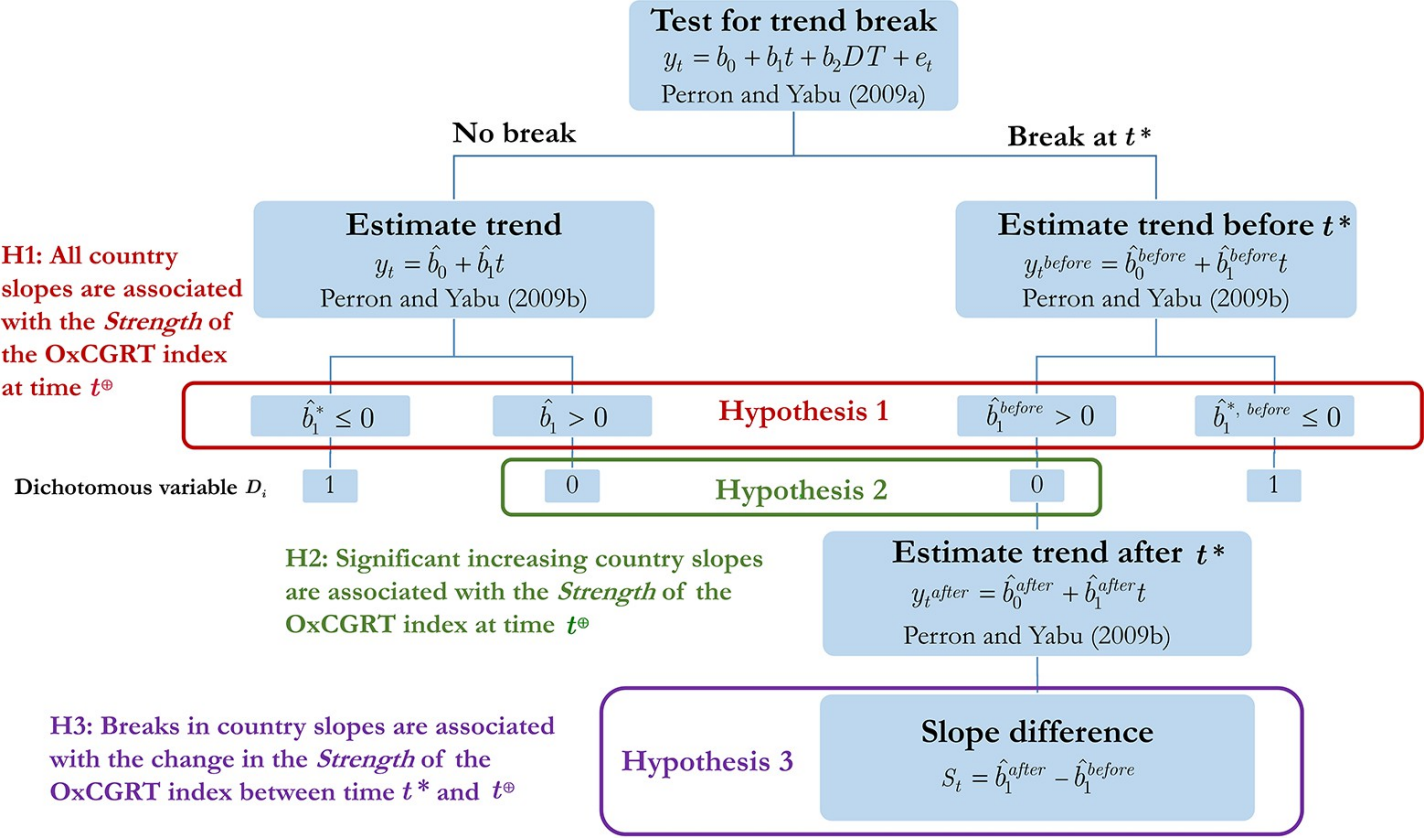
Empirical strategy for investigating the hypothesis 1, 2 and 3.

To assess the overall effectiveness of the conducted policies in response to the COVID-19 outbreak, we follow the empirical strategy deployed in Fig 3 below. As the presence of an unknown structural change severely affects the estimation of the slope on the trend component in a series, we begin our analysis by testing for a trend break based on the Perron and Yabu [10] methodological framework.

Starting from the entire data sample T (1 January 2020 to 30 April 2020), we define for each country i (i = 1,…, 32) the *Effective Testing Sample* ($ETS_{i}$) as the period marked by the first day ($t^{⊕}$) in which the number of confirmed deaths is greater or equal to 5, up to the end of the sample T; that is [$t^{⊕}$, T] (in the case of Italy, for instance, the ETS spans from 27 February 2020 to 30 April 2020). Hence, based on the $ETS_{i}$ and the logarithmic transformation of the confirmed number of deaths for each country (the estimated coefficients (slope) represent the average growth rate of deaths), we test for a break in trend. The results are reported in Table 1.

**Table 1:** Trend break test [10] and slope estimates [11].

Country	Column number					
	(1)	(2)	(3)	(4)	(5)	(6)
	$Exp‐W_{FMS}$	$t^{*}$	${\hat{b}}_{1}$	${\hat{b}}_{1}^{before}$	${\hat{b}}_{1}^{after}$	${\hat{b}}_{1}^{after}‐{\hat{b}}_{1}^{before}$
Argentina	000.965***	-	-0.021***	-	-	-
Austria	000.229***	-	-0.008***	-	-	-
Belgium	016.372***	03-Apr-20	-	0.191***	-0.060***	-0.250
Brazil	012.659***	04-Apr-20	-	0.174***	-0.069***	-0.105
Canada	037.402***	09-Apr-20	-	0.182***	-0.048***	-0.134
Chile	000.235***	-	-0.023***	-	-	-
China	126.144***	12-Feb-20	-	0.145***	-0.082***	-0.227
Denmark	000.380***	-	-0.005***	-	-	-
Egypt	000.221***	-	-0.047***	-	-	-
France	181.331***	30-Mar-20	-	0.198***	-0.025***	-0.223
Germany	068.894***	03-Apr-20	-	0.203***	-0.007***	-0.197
Greece	000.670***	-	-0.022***	-	-	-
Indonesia	002.654***	15-Apr-20	-	0.044***	-0.032***	-0.075
Iran	091.594***	18-Mar-20	-	0.163***	-0.012***	-0.175
Ireland	013.458***	15-Apr-20	-	0.074***	-0.031***	-0.105
Israel	000.201***	-	-0.014***	-	-	-
Italy	154.001***	19-Mar-20	-	0.212***	-0.010***	-0.221
Japan	000.714***	-	-0.030***	-	-	-
South Korea	000.573***	-	-0.001***	-	-	-
Malaysia	000.263***	-	-0.027***	-	-	-
The Netherlands	013.139***	27-Mar-20	-	0.216***	-0.031***	-0.247
Norway	000.238***	-	-0.006***	-	-	-
Panama	000.306***	-	-0.028***	-	-	-
Philippines	002.987***	12-Apr-20	-	0.081***	-0.035***	-0.116
Portugal	017.351***	05-Apr-20	-	0.105***	-0.019***	-0.124
Saudi Arabia	000.261***	-	-0.007***	-	-	-
Spain	374.278***	25-Mar-20	-	0.306***	-0.028***	-0.334
Sweden	013.951***	06-Apr-20	-	0.149***	-0.049***	-0.198
Switzerland	100.086***	01-Apr-20	-	0.121***	-0.052***	-0.173
Turkey	011.668***	05-Apr-20	-	0.185***	-0.006***	-0.179
UK	023.850***	03-Apr-20	-	0.216***	-0.002***	-0.215
US	022.376***	02-Apr-20	-	0.234***	-0.024***	-0.210

*Notes*: For column 1, ***, ** and * denote a statistically significant break in trend at the 0.01, 0.05, and 0.10 level, respectively. For columns 3, 4, and 5, ***, ** and * refer to the statistical significance of the estimated slope.

Table 1 (see column 1) reports the $Exp‐W_{FMS}$ test statistic which detects a statistically significant break in the trend for 19 out of 32 countries. The test results can be used to separate the countries into two distinct groups. The first group includes countries with no significant break in the trend of deaths while the second group consists of countries with a significant break. Therefore, the first group includes 13 countries and the second group 19 countries. For the latter group of countries, the Perron and Yabu [10] test identifies endogenously the break date ($t^{*}$) at which the structural change in trend takes place (see column 2 of Table 1). Unsurprisingly, the date of the break occurs much earlier in China (the origin of the virus). The date of the break in Italy precedes the ones in Spain, France, the US, Germany and the UK (For the UK, the structural break in the trend of deaths comes later than for other European countries including Italy, France, Spain and the Netherlands. Richard Horton (Editor of medical journal The Lancet) has been very critical of the UK’s response to the crisis. For instance, Horton told the Science Select Committee of UK MPs in late March 2020 that the government’s scientific advisers failed to consider early warnings of the seriousness of the epidemic in China). For countries like South Korea or Greece, we do not detect a trend in deaths; these are countries that have reported deaths of a similar magnitude on daily basis for the investigated sample.

Once the significant break date $t^{*}$ has been identified, we proceed by estimating the coefficient of trend component on the series through the method of Perron and Yabu [11]. For the first group of countries, the slope estimation (${\hat{b}}_{1}$) takes place for the entire $ETS_{i}$ (see column 3 of Table 1), while for the second group of countries, the slope estimation (${\hat{b}}_{1}^{before}$) is performed from $t^{⊕}$ (i.e. the start of $ETS_{i}$) up to the identified break date $t^{*}$ (see column 4 of Table 1). Thus, for both groups of countries, we obtain estimates for the average growth rate of deaths (along with their statistical significance) without allowing the identified structural change in the trend to affect the magnitude of the coefficients. Notice, from column 4 of Table 1 that, among those 19 countries with a break in the trend of deaths, Spain records the highest average growth rate of deaths (that is, 30.6% per day) followed by the US (that is, 23.4% per day). After the break, the same 19 countries experience an average growth rate of deaths, which either slows down significantly or turns negative.

Focusing on the significance of the slope estimates (see columns 3 and 4 of Table 1) for all countries, we move on to investigate within a binary choice framework the validity of the first hypothesis. Thus, for each country i (i = 1,…, 32) we define the dichotomous variable $D_{i}$ which takes the value of one (1) if a non-significant slope coeffient is estimated and zero (0) if the coefficient is significant (notice that the slope estimates for the countries with a break are all positive and statistically significant). In this case, $D_{i}$ takes the value of 1 for 12 countries and the value of 0 for 20 countries.

Moreover, for each observation t of the sample T, we define the *strength* ($S^{t}$) of the overall policies (implemented to control the spread of COVID-19) as the average value of the OxCGRT index over the period t−1 to t−14 (The *strength* is calculated for the observations that belong to the [15,T] interval). We further define the concept of *early stage* policies at time t, when t is less than or equal to $t^{⊕}$ (t≤$t^{⊕}$). Thus, for each country i, we build the *early stage strength* of the overall policies at time $t^{⊕}$ (start of $ETS_{i}$), denoted by $S_{i}^{t^{⊕}}$. Hence, the first hypothesis is examined by the following simple probit specification:

$$D_{i}=\vartheta_{0}+\vartheta_{1}S_{i}^{t^{⊕}}+u_{i},$$ (10)

where $\vartheta_{0}$ and $\vartheta_{1}$ are parameters to be estimated and $u_{i}$ is the error term assuming the usual properties (an *i*.*i*.*d*. normally distributed process). A statistically significant and positive value for the coefficient $\vartheta_{1}$ would indicate that the *strength* of the policy measures at an *early stage* ($S_{i}^{t^{⊕}}$) is related positively to the probability of attaining an insignificant increasing slope for the series of deaths. The motivation for using Eq (10) relates to the epidemiological model reported in section 3. In particular, if, at the early stage of an outbreak, anti-contagion policies are implemented, then the transmission rate parameter (β) as well as the growth rate of infections and the growth rate of deaths should all be influenced negatively. Hence, we expect for countries that have implemented harsh anti-contagion policies, the transmission rate to decrease in magnitude and, therefore, the slope of deaths (average growth rate) not to be statistically different from zero. On the other hand, consider the case where such policies are not implemented and/or stringency is low. Then, the transmission rate will increase in magnitude and, therefore, the slope of deaths (average growth rate) will be positive and statistically different from zero. The estimates of Eq (10) are shown in Table 2.

**Table 2:** Probit regression results for $D_{i}=\vartheta_{0}+\vartheta_{1}S_{i}^{t^{⊕}}+u_{i}$ (Hypothesis 1).

Coefficient	Estimate	Marginal effect	Coefficient robust s.e.		Coefficient *p*-value	Coefficient 95% Conf. Interval
$\vartheta_{0}$	-3.571***	-	0.868		0.000	[-5.272, -1.871]
$\vartheta_{1}$	-0.072***	0.011***	0.021		0.000	[-0.032, -0.114]
Statistics and Diagnostics						
McFadden R-squared		-0-10.572		HL Stat. *p*-value		10.447
Log likelihood		-01-9.056		Hetero. LM test *p*-value		10.626

*Notes*: The symbol *** implies that the estimate is significant at the 0.01 significance level. The specification is estimated by the Maximum Likelihood estimation approach using robust standard errors (s.e.). The Hosmer and Lemeshow [18] HL statistic fails to reject the null hypothesis that the observed and expected proportions are the same across all constructed groups, implying a good model fit. Finally, the LM test for heteroscedasticity as illustrated in Davidson and MacKinnon [19] fails to reject the null hypothesis of no heteroscedasticity.

In terms of the first hypothesis (that is, speed is of the essence), the relevant coefficient $\vartheta_{1}$ is positive and significant at the 0.01 significance level. This finding implies that the higher the *strength* of the policy measures at an *early stage*, the higher the probability of attaining an insignificant increasing trend slope for the observed deaths. In more detail, the associated marginal effect to the estimated coefficient (that is, 0.011) suggests that for every unit increase in the *strength* of the OxCGRT index, the probability of attaining an insignificant increasing trend component on the death series increases by 1.1%.

**Fig 4:**
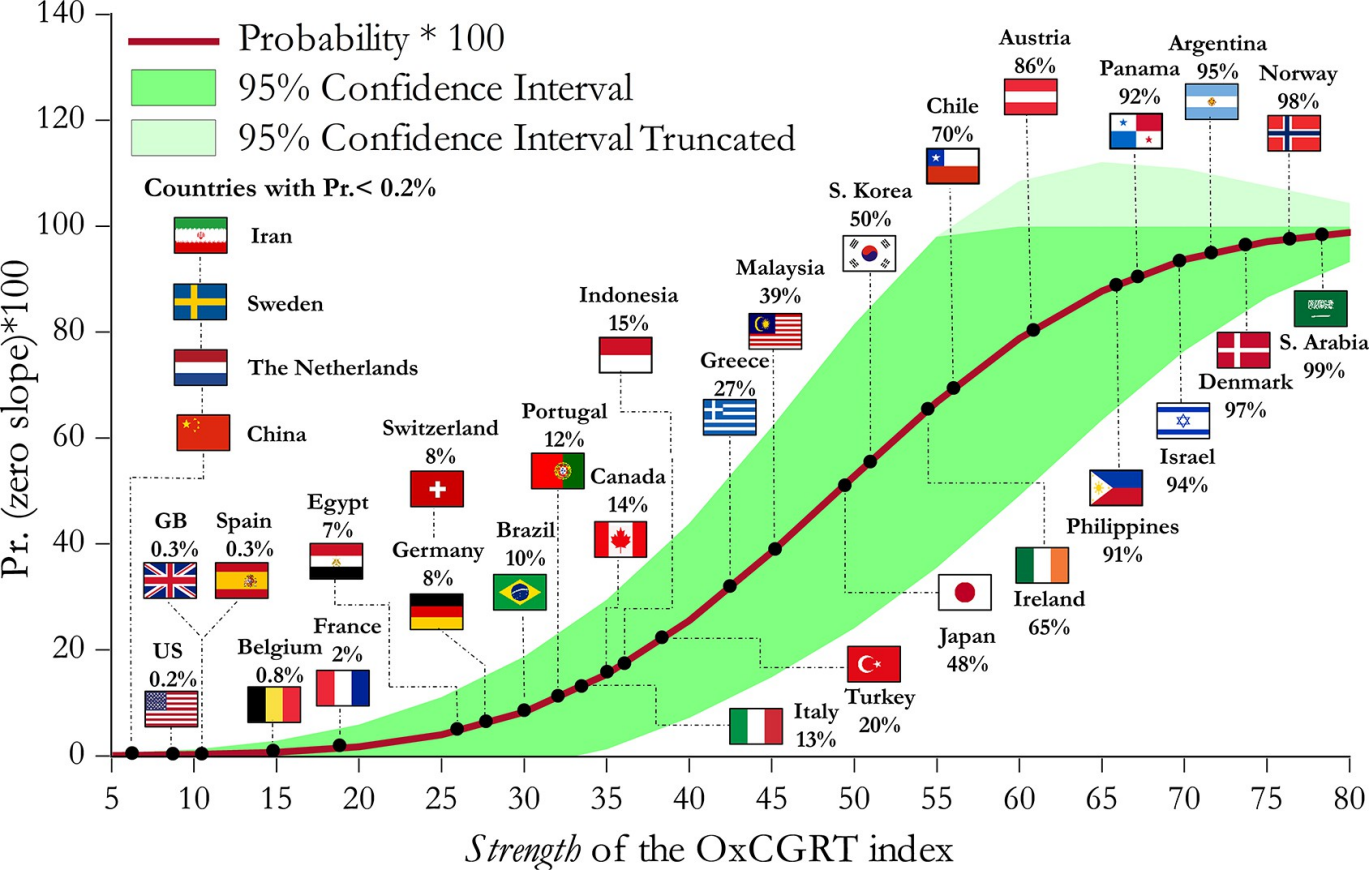
OxCGRT index *strength* at $t^{⊕}$ and probability of insignificant increasing trend.

Fig 4 illustrates, for different *strength* levels of the OxCGRT index at time $t^{⊕}$, the predicted probability (along with the 95% confidence interval) of attaining an insignificant increasing trend component (the predicted probability of attaining an insignificant positive trend for each country along with the respective adult mortality rate indicator per 1000 population are analytically reported in Table A in S1 File)

From Fig 4, policy measures taken at an *early stage* appear to be more effective when their *strength* is greater. For instance, if the *strength* of measures is above 65 units, the predicted probability for attaining a zero slope exceeds 85%, whereas if the *strength* of measures is 35 units, the respective probability is just 15%. We have super-imposed in Fig 4 all countries of our sample. Given, for instance, the *strength* of measures in the U.K., France and Italy, their predicted probabilities are only 0.3%, 2% and 13%, respectively. For South Korea, the predicted probability quadruples to 50% given the country’s greater *strength* of measures at an *early stage*. Overall, our findings offer support to the validity of the first hypothesis.

To examine the validity of the second hypothesis (that is, stringency matters) our focus turns to those 20 countries with a statistically significant positive trend coefficient (see columns 3 and 4 of Table 1). To do so, we construct the $C_{j}^{+}$ variable by assigning to each country j (j=1,…,20) the respective slope (${\hat{b}}_{1}$ or ${\hat{b}}_{1}^{before}$), only if this slope is positive and statistically significant. Thus, the second hypothesis is examined by the following specification:

$$C_{j}^{+}=\mu_{0}+\mu_{1}S_{j}^{t^{⊕}}+u_{j},$$ (11)

where $\mu_{0}$ and $\mu_{1}$ are parameters to be estimated and $u_{j}$ is the error term. A negative value for the coefficient $\mu_{1}$ would indicate that the higher the *strength* of the policies at an *early stage*, the lower the growth rate of deaths for the subsequent period. The estimates of Eq (11) are reported in Table 3 (the motivation for this specification links directly to Eq 6 of Section 3).

**Table 3:** Regression results for $C_{j}^{+}=\mu_{0}+\mu_{1}S_{j}^{t^{⊕}}+u_{j}$ (Hypothesis 2).

Coefficient	Estimate	Newey-West s.e.		*t*-statistic	*p*-value	95% Conf. Interval
$\mu_{0}$	-0.216***		0.025	-8.60	0.000	[-0.171, -0.261]
$\mu_{1}$	-0.002***		0.001	-3.68	0.001	[-0.003, -0.001]
Statistics and Diagnostics						
R-squared			0.295	B.G. Chi-squared *p*-value		0.501
White Chi-squared *p*-value			0.588	J.B. normality *p*-value		0.870

*Notes*: The symbol *** implies that the estimate is significant at the 0.01 significance level. The specification is estimated by OLS using the Newey and West general covariance estimator for the reported standard errors (s.e.). The *p*-value associated to the White’s test for heteroscedasticity implies no heteroscedasticity. Similarly, the *p*-value of the Breusch and Godfrey (B.G.) test for serial correlation suggests no serial correlation. Finally, the *p*-value for the Jarque-Bera test provides evidence in failing to reject the null hypothesis, implying normality for the error term. The diagnostic tests suggest no evidence of specification error.

With reference to the second hypothesis, the relevant coefficient $\mu_{1}$ is negative and significant. The estimated coefficient (-0.002) suggests that for every unit increase in the *strength* of the index at an *early stage*, the slope of the trend component reduces by 0.2%. In the case of the UK, given the *strength* of the country’s measures at $t^{⊕}$, the predicted daily average growth rate of deaths is 19.4% (that is, 0.216–0.002*11; this compares with an actual value of 21.6% from column 4 of Table 1). For Italy, the respective prediction is 14.8% (this compares with an actual value of 21.2% from column 4 of Table 1). Overall, our findings provide support to the validity of the second hypothesis.

For the third hypothesis (that is, speed of adjustment matters), attention is paid to those 19 countries with a significant break in trend (see column 1 of Table 1). We estimate for each country m (m = 1,…, 19) the trend slope after (${\hat{b}}_{1,m}^{after}$) the identified break date through the method of Perron and Yabu [11]. Given our estimates for ${\hat{b}}_{1,m}^{after}$ (see column 5 of Table 1), and ${\hat{b}}_{1,m}^{before}$ (see column 4 of Table 1), we construct the difference ${\hat{b}}_{1,m}^{after}‐{\hat{b}}_{1,m}^{before}$ (see column 6 of Table 1). Moreover, we calculate for each country m the *strength* of its policies at $t^{*}$ denoted by $S_{m}^{t*}$. Hence, the difference of $S_{m}^{t*}‐S_{m}^{t^{⊕}}$ can be seen as the adjustment in the *strength* of the conducted policies over the time distance between $t^{⊕}$ and $t^{*}$ in response to the COVID-19 outbreak. Thus, the final hypothesis is explored through the following specification:

$${\left({\hat{b}}_{1,m}^{after}‐{\hat{b}}_{1,m}^{before}\right)}_{m}=\gamma_{0}+\gamma_{1}{\left(S_{m}^{t*}‐S_{m}^{t^{⊕}}\right)}_{m}+u_{m},$$ (12)

where $\gamma_{0}$ and $\gamma_{1}$ are parameters and $u_{m}$ is the error term. A negative value for $\gamma_{1}$ would indicate an inverse relationship between the adjustment in the *strength* of policies (over the time distance from $t^{⊕}$ to $t^{*}$), and the change in the slopes before and after $t^{*}$. The estimates of Eq (12) are reported in Table 4 (the motivation for this specification links directly to Eq 6 of Section 3 in first differences). With reference to the third hypothesis (speed of adjustment matters), the $\gamma_{1}$ coefficient is negative and significant. Consequently, an increase in the *strength* of measures (between $t^{⊕}$ and $t^{*}$), leads to a decrease in the average growth rate of deaths after $t^{*}$ (compared to the respective growth rate before $t^{*}$).

**Table 4:** Regression results for ${\left({\hat{b}}_{1,m}^{after}-{\hat{b}}_{1,m}^{before}\right)}_{m}=\gamma_{0}+\gamma_{1}{\left(S_{m}^{t*}-S_{m}^{t^{⊕}}\right)}_{m}+u_{m}$ (Hypothesis 3).

Coefficient	Estimate	N.W. s.e.	*t*-statistic		*p*-value	95% Conf. Interval
$\gamma_{0}$	-0.039***	0.049	-0.803		0.434	[-0.134, -0.056]
$\gamma_{1}$	-0.003***	0.001	-3.544		0.002	[-0.005, -0.001]
Statistics and Diagnostics						
R-squared		0.488		B.G. Chi-squared *p*-value		0.708
White Chi-squared *p*-value		0.969		J.B. normality *p*-value		0.592

*Notes*: See the respective notes in Table 3. Overall, the diagnostic tests of the estimated equation suggest no evidence of specification error.

The estimated coefficient (-0.003) suggests that for every unit increase in the *strength* of the index between $t^{⊕}$ and $t^{*}$, the slope in the period after $t^{*}$ reduces by 0.3% (compared to the respective slope for the period before $t^{*}$). In the case of the UK, for instance, where the change in the *strength* of measures is 58.4 units, the predicted change in the daily growth rate of deaths is -21.4% (that is, -0.039–0.003*58.4; this compares with an actual value of -21.5% from column 6 of Table 1). The respective prediction for Italy is -17.9% (this compares with an actual value of -22.1% from column 6 of Table 1).

Overall, by exploiting the trend signals of the death series for 32 countries and relying on the stringency of the conducted policies, we find that government interventions are effective in slowing down or reversing the growth rate of deaths. The studies of Flaxman *et al*. [20] and Hsiang *et al*. [6] reach qualitatively similar findings by implementing different methodological approaches. Flaxman *et al*. [20] fit a counterfactual epidemiological model to 11 European countries to find that strong anti-contagion policy measures have a large impact on reducing transmission. Hsiang *et al*. [6] fit a reduced form econometric model to six countries to find that strong anti-contagion policies have significantly slowed the growth rate of infections.

## 6. Robustness checks and limitations

Published COVID-19 data (registered infections, hospitalizations, deaths) are subject to all kinds of under-reporting. Raftery *et al*. [21] discuss in detail specific limitations and cautions regarding COVID-19 data (cases, hospitalizations, emergency department visits, deaths, excess deaths) and what the implications are for decision making. Indeed, decision makers can make better choices when they have better understanding of the strengths and limitations of these data. Raftery *et al*. [21] note that the number of confirmed COVID-19 cases is “likely to be a substantial underestimate of the prevalence of the disease in a population given that most people with COVID-19 are asymptomatic, and even among those who are symptomatic, not all are tested.” They also note that COVID-19 deaths “are affected by the accuracy of cause-of-death determinations and reflect the state of the outbreak several weeks previously because of the long course of COVID-19 infection.” They view excess deaths as the best mortality indicator of the COVID-19 outbreak, but also note that because of the possibility of death misclassification, excess deaths represent a mix of confirmed COVID-19 deaths and deaths from other causes.

Thus, the presence of measurement errors raises the issue of whether one can be sufficiently confident that the empirical results reported above are reliable to inform decision making. Since our empirical setting focuses on the inherent trend signal of the deaths attributed to COVID-19, our analysis could be potentially misleading if the employed dataset fails to reveal the true trajectory of the trend. To address this concern and following Raftery *et al*. [21] who note that “compared with the other data reviewed here, excess deaths are the best indicator of the mortality impacts of the pandemic”, we look at the excess mortality data compiled by The Financial Times (The FT dataset is provided at https://bit.ly/3G1VCsv. For presentation purposes, missing observations are interpolated through the quadratic-match average method. Again, for presentation purposes, excess deaths are plotted since 2019. Doing so, we see how excess mortality has evolved prior to and during the COVID-19 outbreak).

**Fig 5:**
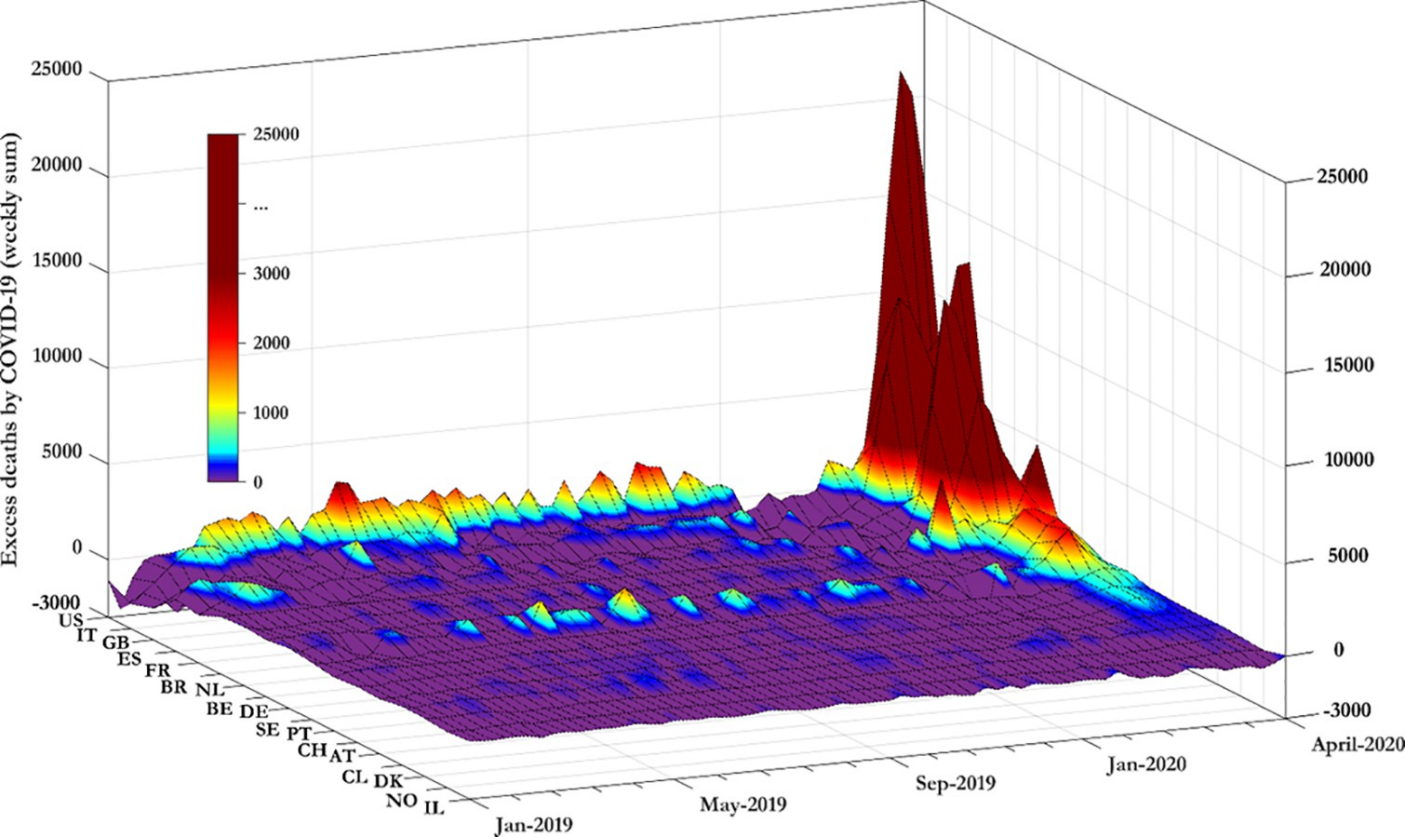
Excess mortality attributed to all causes of death by country.

**Fig 6:**
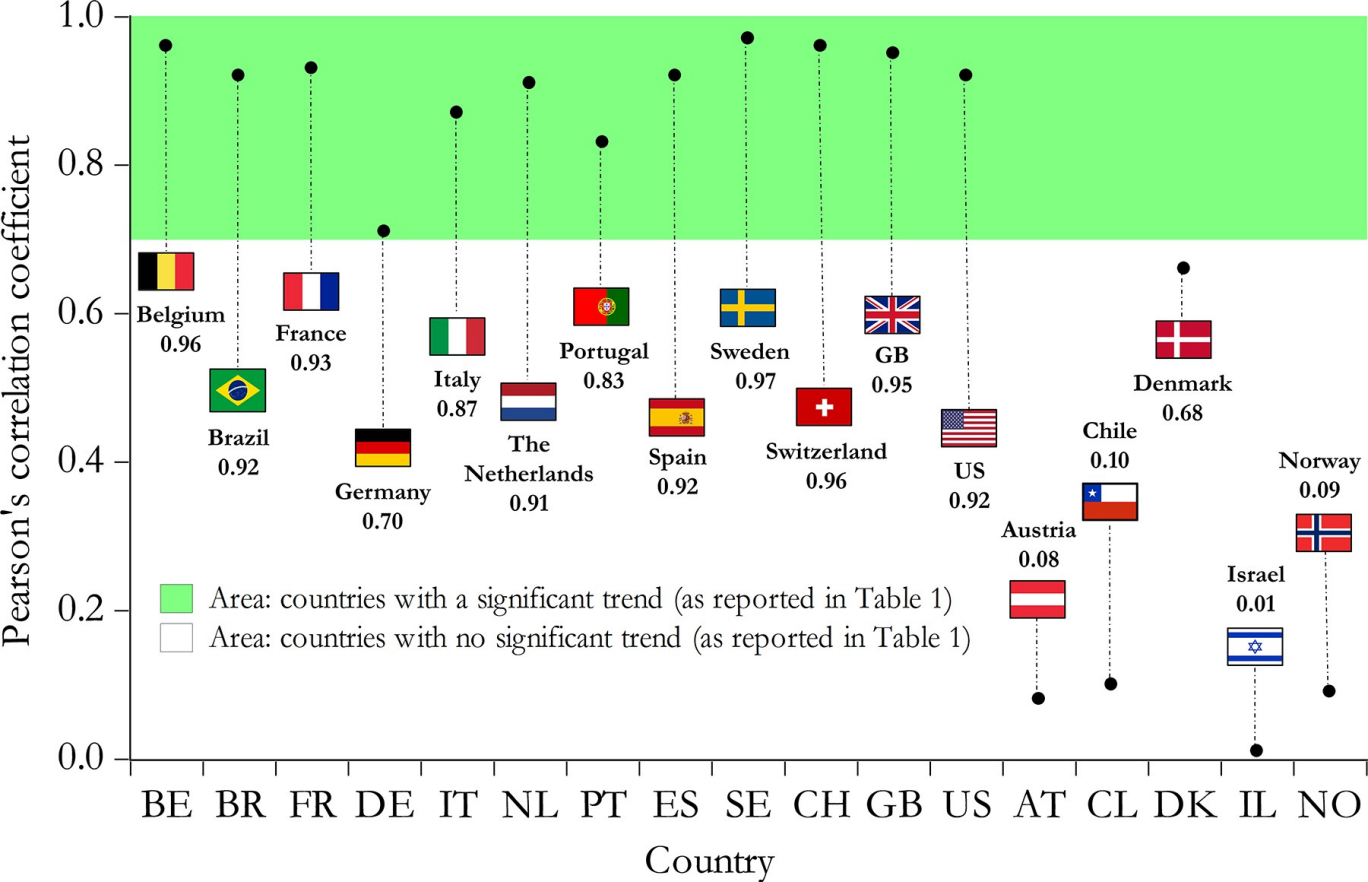
Correlation coefficient between COVID-19 deaths and excess mortality.

The FT dataset provides weekly data on excess mortality for a number of countries (the data for Indonesia and Turkey refer only to Istanbul and Jakarta, respectively, and thus are excluded). Excess mortality refers to the difference between the observed number of deaths (attributed to all causes) in the weeks of January 2019 to April 2020 and the median value for the same periods between 2015 and 2019. Excess mortality data are presented in Fig 5. We focus on 17 countries that are common to the FT excess mortality dataset and our sample; 12 of these countries exhibit positive excess mortality from mid-March 2020 onwards. Although not all of these excess deaths are necessarily attributable to the COVID-19 disease, we may argue with reasonable confidence that excess deaths adequately capture the trend signal of the true COVID-19 deaths. To validate our analysis, we calculate the correlation coefficient between the number of COVID-19 deaths reported by the ECDC dataset and the excess mortality figures reported by the FT dataset (given that our analysis is based on the correct extraction of the underlying linear trend, and by assuming that the excess mortality series for a given country is capable to reveal the actual trend, the correlation coefficient is an adequate statistic to measure the strength of linear association). The correlation coefficients for each country are reported in Fig 6 (for each country, the sample used to estimate the correlation coefficient extends from the time where the first death is observed to 30 April 2020).

We can reasonably expect that COVID-19 deaths from the ECDC dataset would exhibit a high degree of linear association with excess mortality FT data for all countries with a significant positive trend, as reported in Table 1. These are 12 countries in total. Conversely, the COVID-19 deaths from the ECDC dataset would arguably exhibit a lower degree of linear association with the excess mortality FT data, for all countries with an insignificant increasing trend (as reported in Table 1). These are 5 countries in total. From Fig 6, the correlation coefficients for the 12 countries (exhibiting a significant trend) range between 0.70 and 0.97 (the average correlation coefficient value for these 12 countries is 0.90). The correlation coefficients for the remaining 5 countries (exhibiting an insignificant increasing trend) are considerably lower (the average correlation coefficient value for these 5 countries is 0.19). Given these findings, we argue that despite the existence of errors in the measurement of the reported deaths of the ECDC dataset, the dataset is still capable of correctly signaling the true trajectory of the trend. Therefore, our analysis is sufficiently reliable to inform decision making.

To this point we need to mention that the accuracy of the death reporting system in each country may result to two different biases. First a bias may come from the incorrect classification of COVID-19 and non-COVID-19 deaths and second from the completeness of death registration with cause-of-death information in each country. Over the first source of bias, that there is—and probably always will be—a significant classification ambiguity between deaths with and deaths from COVID-19, given that the virus is prone in exacerbating pre-existing conditions [22]. Towards this direction, it seems that there is not much left to be done in terms of working with more accurate data and as such this source of bias should be clearly mentioned as an inherent limitation of the data. Regarding the second source of bias, to gain a sense of its magnitude, across the 32 countries included in the analysis, we collect data from the World Health Organization (W.H.O) database for the completeness of death registration with cause-of-death information (%). This index shows the estimated percentage of deaths that are registered with their cause of death information in the respective registration system of each country. Thus, the lower the completeness of death registration, the higher the error in the reported deaths. For the 31 countries where data are available (no data exist for Indonesia) the completeness of death registration illustrates an average equal to 97%, while for 21 countries the index is equal to 100%. These high values imply that the magnitude of this source of bias is of low importance (the raw data of the completeness index for each country is shown in Table B in S1 File).

We move on and turn our attention to school closures. Rather than using the aggregate stringency index of government interventions, we repeat our analysis by focusing on the ‘school closures’ component of the OxCGRT index. Consequently, we look at the *strength* of the school closure policy defined as the average value of the ‘school closures’ sub-index for the preceding 14-days of the time points of interest (that is; $t^{⊕}$ and $t^{*}$). School closures have been the focus of media attention. The Guardian, for instance, notes that there is very little evidence that school closures are effective in combating COVID-19. Viner *et al*. [23] provide a comprehensive review (see also references therein) of the impact of school closures during coronavirus outbreaks around the world. They make the point that “the evidence to support national closure of schools to combat COVID-19 is very weak and data from influenza outbreaks suggest that school closures could have relatively small effects on a virus with COVID-19’s high transmissibility and apparent low clinical effect on school children” [23]. Viner *et al*. [23] also flag the economic costs of school closures. Indeed, from previous virus outbreaks, school closure costs are estimated between 0.2% and 1% of UK national gross domestic product per annum for school closures of 12–13 weeks and up to 3% of GDP for an 8-week school closure in the US. Ferguson *et al*. [24] use an epidemiological model to find that school closures alone would prevent only 2–4% of deaths, much less than other social distancing interventions.

Using the school closures sub-index in our specifications instead of the aggregate stringecy index, we estimate $\vartheta_{1}$ at 0.034 in Eq (10), $\mu_{1}$ at -0.001 in Eq (11) and $\gamma_{1}$ at -0.001 in Eq (12). These are statistically significant at the 0.01 level but lower in magnitude than the estimates for the aggregate stringecy index. Using school closures, the associated marginal effect to the estimated slope coefficient in Eq (10) is 0.007. This suggests that for every unit increase in the *strength* of school closures sub-index at an *early stage*, the probability of attaining an insignificant increasing trend component on the death series increases by 0.7%. Using school closures, the estimated slope coefficient (-0.001) in Eq (11) suggests that for every unit increase in the *strength* of the school closures sub-index at an *early stage*, the slope of the trend component on the death series reduces by 0.1%. Using school closures in Eq (12), the estimated coefficient (-0.001) suggests that for every unit increase in the *strength* of the school closures sub-index between the time points $t^{⊕}$ and $t^{*}$, the slope in the period after $t^{*}$ reduces by 0.1% compared to the respective slope for the period before $t^{*}$. Therefore, our results suggest that school closures have an impact in driving COVID-19 deaths down on their own but, unsurprisingly, their impact is less powerful compared to the case where a number of policy interventions are combined together. Finally, relying on the findings of Chudik *et al*. [7], rather than using a 14-day window to calculate the *strength* of the conducted policies, we have repeated the entire analysis adopting a 21-day window. The results (available on request) are qualitatively similar to those reported here.

Moreover, we need to mention that disease outbreaks may die on their own in specific geographic regions because of the consumption of all susceptible population. Our analysis minimizes the effects of this concern, by concentrating on the very beginning of the COVID-19 outbreak. Therefore, we may argue that at a country level the susceptible individuals in the limit approximate the population. Thus, the consumption of all susceptible population is not a matter of concern. Of course, at some very specific sub-national regions, one may still argue that the consumption of all susceptible population is rapid, and the outbreak might die out on its own. Such heterogeneity leads to different disease transmission rates in different sub-national regions. While such a disaggregated analysis could result to a more precise statistical inference on the effectiveness of the anti-contagion measures, data availability is a major issue, and an extension towards this direction can be seen as a limitation of our analysis.

Finally, it is true that during the first COVID-19 wave, countries were gearing up their testing, monitoring, and reporting capacity, thus generating valid questions about the quality of the reported data. However, the extension of our analysis to the second wave period involves one major obstacle; the roll out of the vaccination program. As our purpose is to investigate the effectiveness of the government imposed anti-contagion policies per se, over the resulted deaths, an ideal time period would be a sample with no “contamination” by other factors that significantly affect deaths (i.e. vaccination, monoclonal antibodies drugs etc.). In fact, given our methodological framework, it is not feasible to disentangle the net effect of the anti-contagion policies in the presence of the vaccination program. As such, despite the limitations of the first wave COVID-19 period in terms of data quality, this period seems to be the most suitable to evaluate the effectiveness of the anti-contagion policies per se.

## 7. Conclusions

COVID-19 developments dominate the news not only because of the challenging social costs and growing numbers of lives lost, but also the economic costs resulting from closures and social distancing measures. Of course, the spread of a virus in the population does not only depend on its ex-ante characteristics but also on the private and public behavioral responses (i.e., the prevalence response elasticity of private and public demand for protection against the virus). In the context of the COVID-19 pandemic given that governments, on average, acted decisively within a short period of time by deploying a set of measures to protect public health (e.g., lockdowns and social distancing polices), the private and public behavioral responses over the prevalence of the disease can be witnessed as overlapping. An obvious result of the observed commonality in the behavioral responses (private and public), is that these are modeled as one inseparable component (see for instance, Atkeson [25]). Consequently, private behavioral responses are masked by the public intervention policies. Moreover, in a very detailed study by Chudik *et al*. [7] it is illustrated, for the case of the COVID-19 pandemic, that the prevalence response elasticity of private demand starts only in the peak of the pandemic and therefore the overall effect in flattening the epidemic curve is negligible. On the other hand, by Chudik *et al*. [7] show that mandating public behavioral responses is extremely effective in reducing COVID-19 infections and related deaths. Thus, in an empirical context, the consideration of both private and public prevalence response elasticities of demand for protection over the COVID-19 virus, reduces in the assessment of the government policies impact on controlling the COVID-19 pandemic.

This paper assesses the quantitative impact of government interventions on COVID-19 first wave deaths. Using daily data for 32 countries and relying on the stringency of the conducted policies, we find that the greater the *strength* of government interventions at an *early stage*, the more effective these are in slowing down or reversing the growth rate of deaths. School closures, on their own, have a significant impact, but they are less powerful in driving deaths down than are combinations of government interventions. Overall, government decisiveness in taking early action is paramount to control the virus. Forman *et al*. [26] also flag the importance of data, information, models, and the processes by which policy-making decisions are made to be available for scrutiny in order to enhance trust and strengthen the response to the pandemic. We hope that governments can use some of the results of this paper to respond to future COVID-19 outbreaks or other pandemics. This is important not least because there is a possibility of further waves of COVID-19 infections and deaths as governments progressively relax their interventions. In fact, a sustainable exit strategy may not be very straightforward. As noted by Anderson *et al*. [27], the implementation of social distancing measures has been politically challenging but technically simple. On the other hand, easing social distancing measures will most likely involve a process of trial and error, especially if there is evidence of further COVID-19 outbreaks emerging.

## Supporting information

S1 File (DOCX)
