# Supplementary material for: Effectiveness of government policies in response to the first COVID-19 outbreak
Source: PLOS Glob Public Health. 2022 Apr 13;2(4):e0000242. doi: 10.1371/journal.pgph.0000242 (PMC10021334; doi:10.1371/journal.pgph.0000242)
Supplement: S1 File — (DOCX) [file pgph.0000242.s001.docx]

**Supporting information**

**Table A.** Probability of insignificant positive trend and adult mortality rate

| Country | |  | Adult mortality rate |  | Prob. of insignificant positive trend |
| --- | --- | --- | --- | --- | --- |
|  | | | | | |
| Argentina | **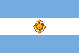** |  | 111.40 | 0.957 | |
| Austria | **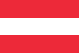** |  | 61.89 | 0.864 | |
| Belgium | **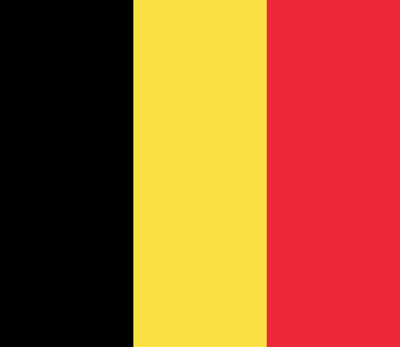** |  | 71.56 | 0.007 | |
| Brazil | **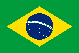** |  | 142.80 | 0.100 | |
| Canada | **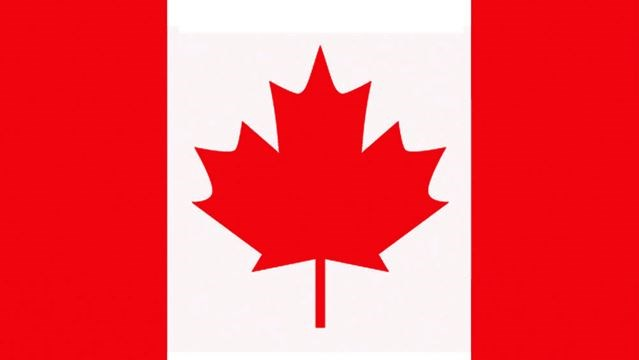** |  | 62.83 | 0.142 | |
| Chile | **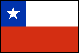** |  | 87.43 | 0.700 | |
| China | **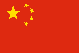** |  | 80.36 | 0.000 | |
| Denmark | **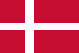** |  | 65.04 | 0.971 | |
| Egypt | **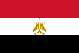** |  | 164.60 | 0.065 | |
| France | **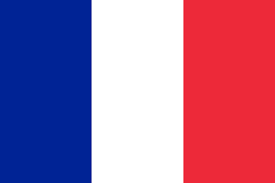** |  | 70.98 | 0.018 | |
| Germany | **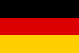** |  | 68.69 | 0.078 | |
| Greece | **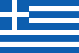** |  | 66.42 | 0.273 | |
| Indonesia | **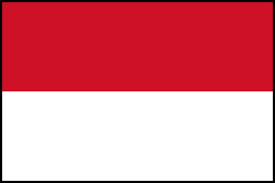** |  | 175.60 | 0.151 | |
| Iran | **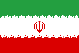** |  | 80.13 | 0.001 | |
| Ireland | **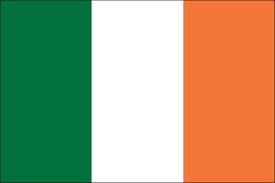** |  | 62.39 | 0.647 | |
| Israel | **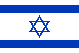** |  | 57.98 | 0.949 | |
| Italy | **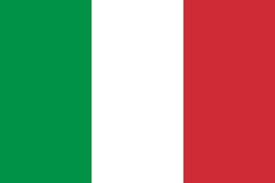** |  | 53.53 | 0.137 | |
| Japan | **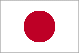** |  | 50.83 | 0.482 | |
| South Korea | **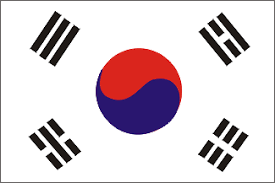** |  | 60.81 | 0.523 | |
| Malaysia | **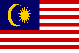** |  | 122.90 | 0.388 | |
| The Netherlands | **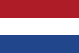** |  | 58.63 | 0.001 | |
| Norway | **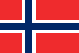** |  | 54.66 | 0.984 | |
| Panama | **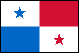** |  | 111.00 | 0.922 | |
| Philippines | **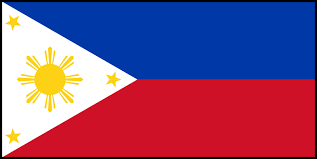** |  | 193.90 | 0.915 | |
| Portugal | **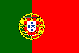** |  | 75.56 | 0.125 | |
| Saudi Arabia | **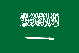** |  | 89.13 | 0.997 | |
| Spain | **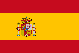** |  | 55.79 | 0.003 | |
| Sweden | **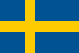** |  | 52.43 | 0.002 | |
| Switzerland | **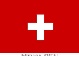** |  | 49.20 | 0.078 | |
| Turkey | **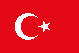** |  | 104.00 | 0.207 | |
| UK | **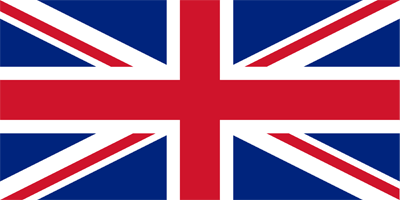** |  | 66.68 | 0.003 | |
| US | **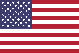** |  | 114.10 | 0.002 | |

*Notes*: i) The adult mortality rate indicator per 1000 population in the first column refers to adults between 15 and 60 years and has been recovered from the World Development Indicators database of The World Bank. ii) the reported probability of attaining an insignificant positive trend, in the second column, is the predicted probability from the estimates of equation (10) and is presented also in Fig 4.

**Table B.** Completeness of death registration with cause-of-death information (%)

| Country | |  | Latest observation year |  | Completeness of death registration |
| --- | --- | --- | --- | --- | --- |
|  | | | | | |
| Argentina | **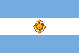** |  | 2016 | 100% | |
| Austria | **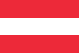** |  | 2017 | 100% | |
| Belgium | **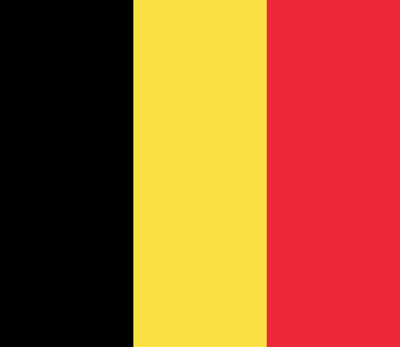** |  | 2016 | 100% | |
| Brazil | **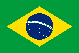** |  | 2016 | 99% | |
| Canada | **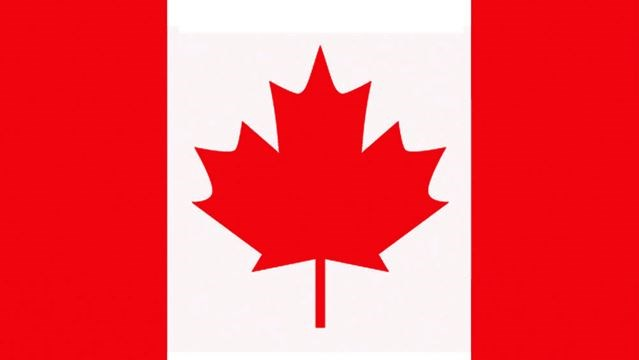** |  | 2015 | 100% | |
| Chile | **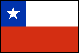** |  | 2016 | 95% | |
| China | **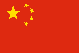** |  | 2015 | 62% | |
| Denmark | **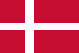** |  | 2015 | 100% | |
| Egypt | **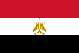** |  | 2015 | 94% | |
| France | **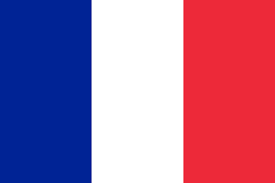** |  | 2015 | 100% | |
| Germany | **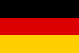** |  | 2016 | 100% | |
| Greece | **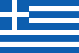** |  | 2016 | 100% | |
| Indonesia | **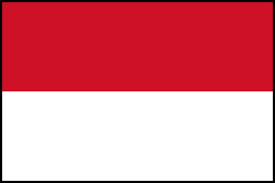** |  | NA | NA | |
| Iran | **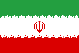** |  | 2016 | 90% | |
| Ireland | **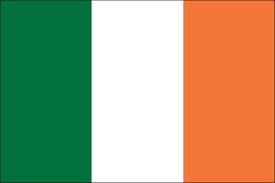** |  | 2015 | 100% | |
| Israel | **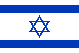** |  | 2016 | 100% | |
| Italy | **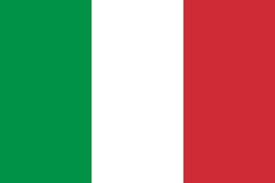** |  | 2015 | 100% | |
| Japan | **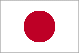** |  | 2016 | 100% | |
| South Korea | **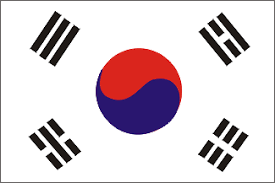** |  | 2016 | 100% | |
| Malaysia | **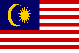** |  | 2016 | 90% | |
| The Netherlands | **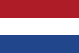** |  | 2016 | 100% | |
| Norway | **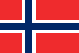** |  | 2016 | 100% | |
| Panama | **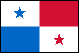** |  | 2016 | 92% | |
| Philippines | **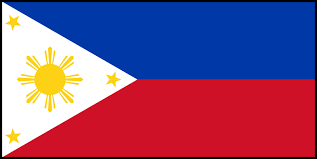** |  | 2011 | 89% | |
| Portugal | **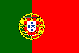** |  | 2016 | 100% | |
| Saudi Arabia | **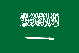** |  | 2017 | 95% | |
| Spain | **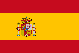** |  | 2016 | 100% | |
| Sweden | **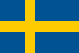** |  | 2016 | 100% | |
| Switzerland | **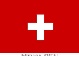** |  | 2016 | 100% | |
| Turkey | **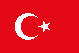** |  | 2016 | 92% | |
| UK | **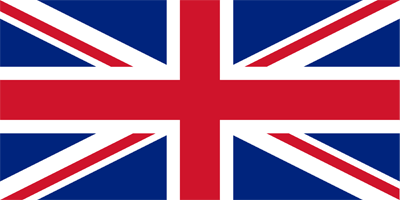** |  | 2016 | 100% | |
| US | **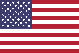** |  | 2016 | 100% | |

*Notes*: i) The completeness of death registration, in the second column, is the estimated percentage of deaths that are registered with their cause of death information in the vital registration system of a country and has been recovered from the World Health Organization database. ii) The first column shows the latest available year where the completeness of death registration reported data refer to.
